# Before Sustainable Development Goals (SDG): why Nigeria failed to achieve the Millennium Development Goals (MDGs)

**DOI:** 10.11604/pamj.2016.24.156.8447

**Published:** 2016-06-22

**Authors:** Obinna Ositadimma Oleribe, Simon David Taylor-Robinson

**Affiliations:** 1 Excellence & Friends Management Care Centre (EFMC), Abuja, Nigeria; 2 Hepatology Unit, Imperial College London, 10th Floor, QEQM Building, St Mary’s Hospital Campus, South Wharf Road, W2 1NY, London, United Kingdom

**Keywords:** Sustainable Development Goals (SDG), Nigeria, Millennium Development Goals (MDGs)

## Abstract

World leaders adopted the UN Millennium Declaration in 2000, which committed the nations of the world to a new global partnership, aimed at reducing extreme poverty and other time-bound targets, with a stated deadline of 2015. Fifteen years later, although significant progress has been made worldwide, Nigeria is lagging behind for a variety of reasons, including bureaucracy, poor resource management in the healthcare system, sequential healthcare worker industrial action, Boko Haram insurgency in the north of Nigeria and kidnappings in the south of Nigeria. The country needs to tackle these problems to be able to significantly advance with the new sustainable development goals (SDGs) by the 2030 target date.

## Commentary

In September 2000, at the Millennium Summit, the world leaders adopted the UN Millennium Declaration, which committed the nations of the world to a new global partnership, aimed at reducing extreme poverty and other time-bound targets, with a stated deadline of 2015 [1]. The Millennium Development Goals (MDGs) therefore have been the world´s only time-bound and quantifiable targets for addressing extreme poverty in its many dimensions: income poverty, hunger, disease, lack of adequate shelter, and social exclusion, while promoting gender equality, education, and environmental sustainability [1]. This eight goal initiative was planned to eradicate extreme poverty and hunger, while aiming to achieve universal primary education, promote gender equality, reduce child mortality, improve maternal health, combat HIV/AIDS, malaria, and other diseases, ensure environmental sustainability and develop a global partnership for development [2]. Based on these goals, the world has galvanized previously unprecedented efforts to meet the needs of the world’s poorest and most disadvantaged [3].

Nigeria, like most other nations of the world, signed this treaty and promised to work towards the realization of this goal. To achieve this, a number of steps were taken, including the release of central government funds. Offices were created and individuals appointed to key positions to work towards the MDG targets.

According to the United Nations, the number of people living in extreme poverty declined worldwide by more than half, falling from 1.9 billion in 1990 to 836 million in 2015; primary school enrolment rate has increased and the number of out-of-school children of primary school age worldwide has fallen by almost half, to an estimated 57 million in 2015, down from 100 million in 2000 across the world. There has been significant improvement in gender equality with empowerment of women as more girls are in school, more women are in paid employment and many more women are now in government around the world. Global under-five year mortality rate has declined by more than half, dropping from 90 to 43 deaths per 1,000 live births between 1990 and 2015 [2]. In addition, maternal mortality ratio has declined by 45 per cent worldwide since 1990 with an improvement in contraceptive prevalence; and new malaria and HIV cases have declined, with new HIV infections falling by approximately 40 percent between 2000 and 2013, from an estimated 3.5 million cases to 2.1million [2].

However, at the end of the MDG period in 2015, where is Nigeria in all this as a nation? There are a number of unsupported claims that Nigeria achieved most of the goals, especially the HIV and maternal mortality MDG targets ahead of deadline [4]. However, according to the UN report, “nearly 60 percent of the world’s 1 billion extremely poor people lived in just five countries in 2011: India, Nigeria, China, Bangladesh and the Democratic Republic of the Congo”[2]. Nigeria contributed significantly to more than 42,000 people who were forced to abandon their homes and seek protection due to armed conflicts in 2014 [2]. Of the 2.1 million new HIV infections that occurred in 2013, 75% occurred in just 15 countries with Nigeria, South Africa and Uganda accounting for almost half of them all [2]. Moreover, Nigeria has one of the lowest number of children sleeping under the mosquito nets, in a comparison of surveys among nations of the world [2].

**Nigeria and the MDGs:** According to recent estimates, Nigeria has an infant mortality rate of 72.7 deaths/1,000 live births, a contraceptive prevalence of 15.1% (2013), health expenditure of 3.9% of GDP (2013); HIV prevalence of 3.17% (2014 est.), a HIV burden of 3,228,600 (2013) and HIV-associated deaths of 174,300 (2014), with life expectancy at birth of 53.02 years [5]. These were not unexpected, as a review of the MDGs in Nigeria by Olabode and colleagues in 2014 concluded that Nigeria would not attain the MDG targets by the end of 2015, even if smaller nations in Africa did so, such as Ghana, Cameroon and Botswana [6]. Nigeria, like most sub Saharan African nations, has failed to meet any of the targets due to a multiplicity of health system-related, political and systemic challenges [7].

The recent claims that Nigeria met some of the MDGs need to be fully supported and validated. Without epidemiological data to the contrary, it is difficult to believe that such milestones have been reached. It is questionable as to whether infant deaths have reduced in Nigeria and if there has been a significant change in malaria-induced mortality and morbidity. It seems that maternal death rates have not significantly changed. With the Federal Government yet to pay teachers’ salaries, whether schools have higher enrolment is a moot point. With the regular healthcare workers’ strikes, attempts to reduce national mortality rates have been severely hampered.

From the current national health outcome reports, it will be a disservice to Nigeria and Nigerians for anyone – government or international organizations- to claim that any of the eight MDG targets were actually met in Nigeria to date. To be politically correct, one can tell the world that the exact situation is unknown currently in Nigeria. This is actually the truth, even though it is unacceptable by all standards. When stated as it is, we will ask ourselves the one-million-dollar question - why have the goals not been met and why is progress made largely unquantified?

In this commentary, the authors will be looking at why Nigeria, like many sub-Saharan African countries, did not meet the MDG targets. This exercise is critical, as it is fundamental to the success or failure of the recently launched Sustainable Development Goals (SDGs), which now take over from the 2000 MDGs [8]. If the factors that made the MDGs fail are not examined and thoroughly, they will likewise cause the colossal failure of SDGs, despite huge investments in human, material and financial resources.

In 2014, Ajiye identified lack of human capacity for implementation, poor access to primary healthcare delivery systems with high cost of healthcare, inadequate and unreliable data systems, inadequate funding and indiscipline with endemic corruption as challenges that were facing MDGs in Nigeria [9]. We agree with Ajiye’s findings and believe that Nigeria did not achieve the targets. The reason, apart from all identified by Ajiye, include (but are not limited to):

***Wrong assumptions:*** the assumptions on which the MDGs were predicated were fundamentally wrong. It was believed that the poor health indices in Nigeria were as a result of poverty and lack of resources. Because of this, central funds were released and injected into the ‘healthcare system’ to overcome these inequalities. It was also assumed that individuals appointed to manage the funds had the requisite qualifications, the interest of the nation and the program at heart, as well as the capacity to manage the funds successfully towards the achievement of the MDG targets. It was also assumed that the systems were in place to support the activities towards the achievement of the MDGs, but this was not the case. The outcomes were far from what was expected.

***Absence of true and validated baseline data:*** Since independence, Nigeria has survived on public health “guesstimates”, rather than informed estimates. There is no single dependable, reliable, validated and easily verifiable public health dataset in Nigeria. Even organisations that ought to have these datasets like the National Health Insurance Scheme (NHIS) do not have a validated, verifiable dataset of those enrolled into the insurance system. All attempts to have national ID cards, proper censuses and nationwide surveys have failed to deliver verifiable results. This account for seemingly “150 - 200%” coverage rates on National Immunization days, even when there are obvious deficiencies in the process. The basis for most calculations and projections are very faulty. MDGs cannot succeed in Nigeria when there are no real baseline data with which to compare progress.

***Absence of formative, midcourse and proper end-line evaluation:*** While a lot of resources were invested into the management of MDGs, little was done in terms of progress (formative), midcourse and end-line (summative) evaluations to effectively and scientifically look at the progress of the roll-out of the MDG program. If these had been done, it would have given the managers early warning signs that the delivery of the MDG program was off-course, and thus, necessitate midcourse corrections. These evaluations, audits and consequent corrections were never carried out. Rather, the program depended on oral reports, informal adhoc data from program managers designed to make the National President and the world happy, as well as positive newspaper reports of opening of new healthcare centres, donation of medical equipment and increased employment of healthcare workers. These were wrong measures of success.

***Sequential healthcare workers’ industrial actions in Nigeria:*** In a recent study, conducted by our organization and presented at the 38th/39th West African College of Physicians Annual General and Scientific Meeting in Abuja, Nigeria, there were more than 10 different healthcare workers’ strikes in Nigeria over a 36-month period. These paralyzed the healthcare industry, resulting in avoidable mortality and morbidities, as well as catastrophic health expenditure and resultant outgoing medical tourism. Children and pregnant women were the worst victims of the healthcare worker industrial action. Without access to affordable healthcare services, deaths were inevitable. Claiming to have reduced mortality and morbidity in Nigeria, therefore, needs detailed and verifiable epidemiological data to the contrary.

***Boko Haram insurgency in the north, and kidnapping in the south:*** The upscale of social discord, killings and bombings in the northern part of Nigeria; and kidnapping in southern Nigeria reversed the gains of so many years of investments in healthcare in Nigeria, especially in affected communities. Today, there are several hundreds of thousands of internally displaced persons who are current victims of communicable diseases, malnutrition and several other social problems. This figure was estimated to be 1,538,982 as of April 2015 by the internally-displaced monitoring centre [10]. As these people live on charity, have limited access to healthcare services, school enrolment and healthy shelter, their health and emotional conditions are far from ideal. These people are also denied access to quality care, even when they could afford it. Sexual exploitation and harassment has led to several unwanted pregnancies and maternal deaths. Fear of attacks has led to mass exodus of healthcare workers, closure of healthcare facilities and deserted communities, resulting in difficulties in accessing healthcare during emergencies, outbreak of communicable diseases, and many avoidable deaths and complications.

***Absence of National Health Insurance Scheme:*** As at mid-2012, NHIS still covered only about 3 percent of the population (that is about 5 million individuals). By the time of this report, less than 6 percent of Nigerians have access to health insurance schemes in Nigeria. Again, this figure is not verifiable, nor is it reliable. People pay for services from out-of-pocket expenditure, accounting for more than 60% of healthcare costs in Nigeria. This results in various types of delays including accessing care, seeking care, receiving care at the health facilities, obtaining prescribed care, and delays in leaving the healthcare facility after treatment has taken place. These delays deepen the physical challenges of the patients and facilitate nosocomial infections, which usually results in additional associated cost of care.

***Verticalization of the healthcare system:*** Vertical programs may deliver immediate positive change, but they are neither effective, nor sustainable. For decades, the world and donor agencies have depended on this strategy, but with the same inadequate results. This was sustained in MDGs. Using the available resources to fight a single disease or group of diseases is programmatically interesting, but economically unsound, as other conditions are often forgotten or under-resourced as a result. This practice fails to enjoy the economy of scale, common with integrated services. Also, it cannot leverage on the competencies and equipment from other disease managers. New personnel, equipment and facilities are built in some cases to accommodate these vertical projects, resulting in increased workload to the inadequately-skilled and already overstretched existing healthcare workers.

Other factors that may have contributed to the failure of the MDG project in Nigeria include poor guidance from the MDGs funders.

**Lessons learnt from the MDGs:** it is clear that facts, not estimates (and at worst, guesstimates) are needed for success of the SDGs. It is necessary to begin to analyse hospital-based data and use them to make meaningful projections. It is important to have multicentre studies that will have good external and internal validity. National agencies also need to publish periodically on their websites, validated data for public consumption.

Second, there should be real healthcare leaders, not managers, as drivers of the SDGs. Medical qualification and years of experience in the health industry may not be enough in the choice of leaders of the SDGs. People and individuals who have the right leadership orientation, skills and competences should be tasked with the assignment of ensuring effective take-off, implementation, evaluation and reporting of the SDGs. SDGs should not be allowed to go the way of the MDGs.

Third, there is the need for full integration rather than verticalization of healthcare services. This will allow for leveraging of resources, development of sustainable processes and healthcare systems, as well as maximization of economies of scale. Besides, it will reduce data load on healthcare workers, prevent monotony in delivery of healthcare services, empower healthcare workers with lateral skills for multitasking and ensure continuity of services, even after 2030. This single healthcare objective seen in SDG will encourage this, if well utilized.

Fourth, proper and measurable process (formative) evaluations are critical at key intervals and should be built into the implementation plans. This will help keep the implementation of the SDGs program on course, and when deviations occur, make corrections early enough to achieve the goals as at 2030. Systems should be developed and put in place in all segments of the health system – including fund management systems. Finally, individuals should be trained and retrained to ensure proper reorientation with a new integrated care mentality. These trainings should also be aimed at building transparency into the system, developing skilled data managers and excellent evaluators who will conduct both the process and summative evaluations. The time to work differently in Nigeria is now. Positive change is a choice, and not a chance occurrence. Change results from choices made, not a product of what is happening. It is triggered by purpose, passion, focus, sacrifices, and discipline. Nigeria must make positive changes to achieve the SDGs come 2030.
